# Health workers' experiences of collaborative quality improvement for maternal and newborn care in rural Tanzanian health facilities: A process evaluation using the integrated 'Promoting Action on Research Implementation in Health Services' framework

**DOI:** 10.1371/journal.pone.0209092

**Published:** 2018-12-19

**Authors:** Ulrika Baker, Arafumin Petro, Tanya Marchant, Stefan Peterson, Fatuma Manzi, Anna Bergström, Claudia Hanson

**Affiliations:** 1 Department of Family Medicine, College of Medicine, University of Malawi, Blantyre, Malawi; 2 Department of Public Health Sciences, Global Health—Health Systems and Policy Research, Karolinska Institutet, Stockholm, Sweden; 3 Department of Neurobiology, Care Sciences and Society, Division of Family Medicine, Karolinska Institutet, Huddinge, Sweden; 4 Ifakara Health Institute, Health Systems, Impact Evaluation and Policy (HSIEP), Dar es Salaam, Tanzania; 5 Department of Disease Control, London School of Hygiene & Tropical Medicine (LSHTM), London, United Kingdom; 6 Department of Women’s and Children’s Health, International Maternal and Child Health (IMCH), Uppsala University, Uppsala, Sweden; 7 Makerere School of Public Health, Kampala, Uganda; 8 UNICEF, Health Section, Programme Division, New York, United States of America; 9 Institute for Global Health, University College London, London, United Kingdom; Medical Research Council, SOUTH AFRICA

## Abstract

**Background:**

Quality Improvement (QI) approaches are increasingly used to bridge the quality gap in maternal and newborn care (MNC) in Sub Saharan Africa. Health workers typically serve as both recipients and implementers of QI activities; their understanding, motivation, and level of involvement largely determining the potential effect. In support of efforts to harmonise and integrate the various QI approaches implemented in parallel in Tanzanian health facilities, our objective was to investigate how different components of a collaborative QI intervention were understood and experienced by health workers, and therefore contributed positively to its mechanisms of effect.

**Materials and methods:**

A qualitative process evaluation of a collaborative QI intervention for MNC in rural Tanzania was carried out. Semi-structured interviews were conducted with 16 health workers in 13 purposively sampled health facilities. A deductive theory-driven qualitative content analysis was employed using the *integrated Promoting Action on Research Implementation in Health services* (i-PARIHS) framework as a lens with its four constructs *innovation*, *recipients*, *facilitation*, and *context* as guiding themes.

**Results:**

Health workers valued the high degree of fit between QI topics and their everyday practice and appreciated the intervention’s comprehensive approach. The use of run-charts to monitor progress was well understood and experienced as motivating. The importance and positive experience of on-site mentoring and coaching visits to individual health facilities was expressed by the majority of health workers. Many described the parallel implementation of various health programs as a challenge.

**Conclusion:**

Components of QI approaches that are well understood and experienced as supportive by health workers in everyday practice may enhance mechanisms of effect and result in more significant change. A focus on such components may also guide harmonisation, to avoid duplication and the implementation of parallel programs, and country ownership of QI approaches in resource limited settings.

## Introduction

The *quality gap* is recognised as a critical limiting factor in accelerating the reduction of maternal and newborn deaths in Sub Saharan Africa [1–3]. This gap implies that while an unprecedented proportion of women seek care for themselves and their newborns during pregnancy, childbirth and the postpartum period, the content of care received is often of insufficient quality to have a significant impact on mortality and morbidity [2, 4].

Various approaches to quality improvement (QI) are used to address this quality gap in Sub Saharan African countries and include for example the “5 S”, “Standards-Based Management and Recognition” and collaborative QI using “Plan-Do-Study-Act” (PDSA) cycles, introduced into routine health care settings [5–9]. These approaches can be seen as *implementation interventions*, in that they aim to increase the use of existing knowledge and its implementation in practice [10]. Health workers typically serve as both *recipients* and *implementers* of these interventions; their understanding, motivation, and level of involvement therefore largely determining their potential impact [11]. In the context of limited resources, where there is a severe lack of skilled health workers and conditions for care provision are unpredictable, the implementation of such interventions may be challenging [12–15].

In Tanzania, the application of QI approaches has been spearheaded by HIV/AIDS programs and in more recent years also used by programs aimed to improve maternal and newborn care [16–19]. The various approaches promote distinct methodologies while often having several *components* in common [6–8]. In practice, different QI approaches may be implemented in parallel in the same district causing duplication, inefficiency and at worst, confusion among health workers [16]. This uncoordinated parallel implementation may impact negatively on the health system’s absorptive capacity, its ability to learn and incorporate new practices, and therefore potentially limit the positive effects of implemented interventions [20]. To share experiences and best practice, a National Quality Improvement Forum has therefore been established with the vision to harmonise the different QI approaches implemented in Tanzania [16]. To support this agenda, more evidence is needed on the role of the different *components* of QI approaches in this context: which aspects are understood and fulfil the perceived needs of health workers in every day practice and therefore contribute positively to the mechanisms of effect, i.e. how the QI interventions produce change [21]?

The EQUIP (Expanded Quality Management Using Information Power) was a collaborative QI intervention targeting community, health facility and district levels, implemented in one rural district in Tanzania and one rural district in Uganda in 2011–2014 [17, 22]. The aim of EQUIP was to increase coverage and quality of a number of essential evidence-based interventions for maternal and newborn care as outlined by WHO and partners [23]. Examples of interventions included promotion of mothers’ preparedness for birth and administration of Oxytocin within 1 minute of childbirth. The latter was one of four primary outcomes for which the outcome evaluation showed a positive effect: an increase of 26% and 8% of the proportion of mothers receiving this intervention in Tanzania and Uganda respectively [24]. This was achieved despite significant contextual challenges, the most notable being poor readiness of health facilities in terms of lack of drugs and equipment and also the project’s limited implementation strength [14, 15, 24, 25]. In Tanzania, improvements were also seen in two locally identified improvement topics: mothers’ preparation of clean birth kits and the frequency of district supervision of lower level health facilities [24].

In this study, we report on a qualitative process evaluation conducted to gain a deeper understanding of how the EQUIP intervention worked in rural health facilities in Tandahimba district in Tanzania. Utilising the recently published *integrated Promoting Action on Research Implementation in Health Services* (i-PARIHS) framework [26] as a lens during analysis, our objective was to investigate how the different components of this collaborative QI intervention were understood and experienced by health workers and therefore, contributed to the mechanisms of effect. The perspective of health workers was chosen in recognition of their central role as both recipients and implementers of the EQUIP intervention.

## Materials and methods

### Study design

This study was a qualitative process evaluation of the EQUIP intervention in health facilities, applying the i-PARIHS framework as a lens during analysis [21, 26].

### The EQUIP intervention in health facilities: logic model and analytical framework

The EQUIP intervention and results have previously been described in detail [17, 24]. Here, we provide a summary of the health facility component with a more detailed description in S1 File.

**Fig 1:**
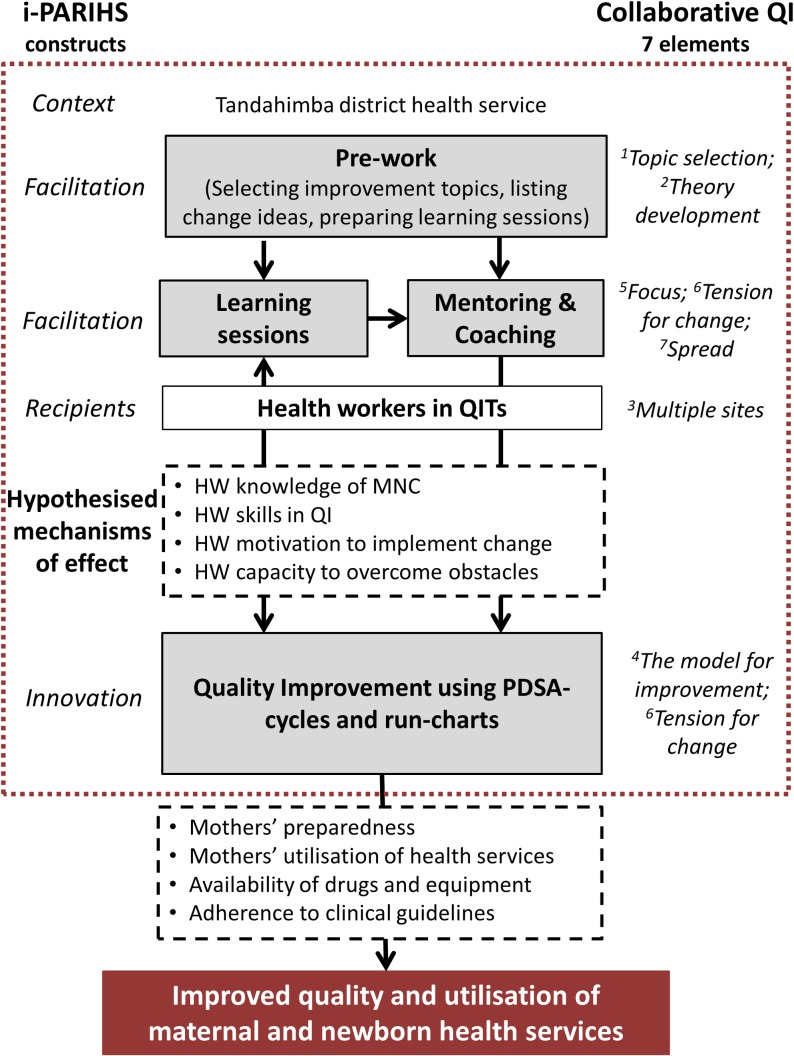
Schematic logic model of the EQUIP intervention in health facilities. The grey-shaded boxes contain the core intervention components [17], with arrows representing the relationship between these. Hypothesised mechanisms of effect are contained within the dashed boxes and the intended outcomes in the red-shaded box. On either side of the logic model are mapped the constructs of the i-PARIHS framework [26] and the seven elements of Collaborative QI which are mapped according to their numbering in the paper by Kilo et al [22]. The red dashed square illustrates the focus for this process evaluation.

EQUIP was modelled on the Institute for Healthcare Improvement’s (IHI) Breakthrough series for collaborative QI, an approach which includes seven elements envisaged to work in synergy to achieve improvement [22]. A schematic logic model, describing the components of the EQUIP intervention, its hypothesised mechanisms of effect and intended outcomes, is outlined in Fig 1. The same figure also illustrates how the elements of collaborative QI and the constructs of the i-PARIHS framework relate to the EQUIP intervention.

The i-PARIHS framework poses four constructs involved in implementation, the characteristics of which will determine its success. These constructs include the *innovation* to be implemented, the *recipients* of this innovation and the *context* in which the innovation is introduced. *Facilitation*, the core construct, is widely defined as a process of enabling, helping or making something easier and is considered to activate implementation through its interaction with the other three constructs [26]. In essence, successful implementation is more likely where an innovation, e.g., a clinical guideline or a triage system, is clearly defined, has a high degree of fit but at the same time a comparative advantage to existing practice [26, 27]. Recipients need to be motivated, have a sufficient level of skills, knowledge, and authority to engage with the innovation in a context with supportive leadership and sufficient resources [28]. The method of facilitation should ensure a high level of participation of key stakeholders, be integrated and iterative and sensitive to the context where it is introduced [28].

Applying the i-PARIHS constructs, the *innovation* introduced by EQUIP can be conceptualised as the use of Plan-Do-Study-Act (PDSA) cycles to structure problem solving and testing change ideas for prioritised improvement topics and tracking progress over time using run-charts. Although the aim of EQUIP was to improve the care provided to mothers and newborns, the primary *recipients* of the innovation were health workers of different cadres, organised into Quality Improvement Teams (QITs) in the 32 public and faith-based health facilities providing maternal and newborn care. The *context* where implementation took place was the district health service in Tandahimba district [17]. *Facilitation* in EQUIP, the process through which implementation of the innovation was enabled, consisted of 3–4 monthly learning sessions for a cluster of health facilities and monthly mentoring and coaching visits to individual health facilities. These activities were carried out by an EQUIP mentor, a member of the EQUIP implementing team, together with a district mentor, one of the members of the Council Health Management Team. Another part of the facilitation in EQUIP, not immediately visible to health workers, was the extensive prework carried out by EQUIP and district mentors, during which improvement topics were prioritised, learning sessions prepared and potential change ideas developed.

The hypothesised mechanisms of effect of EQUIP can be divided into two stages. In the first stage, the hypothesis is that learning sessions together with mentoring and coaching lead to increase in health workers’ knowledge and skills of the improvement topics and also their motivation and capacity to implement QI using PDSA-cycles and run-charts. In the second stage, this implementation of QI would lead to improved quality of care provided for mothers and newborns in the health facilities, through for example improved procurement of drugs and supplies and increased adherence to clinical guidelines.

### Study setting

EQUIP was implemented in Tandahimba district in Mtwara region in south-east Tanzania. Characteristics of the district have been reported on in detail elsewhere [14, 29]. Briefly, the population of approximately 200,000 is predominantly subsistence farmers and serviced by one district hospital, three health centres and 29 dispensaries, all except one providing maternal and newborn care. At the time of our study, several external partners were involved in implementing various health programs in the district health facilities. Challenges for care provision include a lack of health workers, drugs and equipment [15]. In a previous paper, we explored health workers’ experiences of the conditions for care provision which were perceived as inherently unpredictable and largely outside of their control [29]. Health workers also expressed often being alone and feeling unsupported in their work [29].

### Data collection

In February 2014, semi structured interviews were conducted with 16 health workers in 13 health facilities in Tandahimba, purposively sampled to reflect all levels of facilities (hospital, health centre and dispensary) and the varying degree to which QITs engaged in the EQUIP intervention, described as their *functionality*. The EQUIP mentors conducted the assessment of this functionality based on rating seven characteristics of the QITs including their knowledge of new improvement topics, PDSA cycles, work plans, change ideas and the use of run-charts (S2 File). Respondents were included from the hospital and all three health centres while ten out of the twenty-eight dispensaries were sampled through a two-stage process. Dispensaries were first sorted according to their caseload of deliveries in the previous month and from those with the highest case load, five facilities with a high functionality of their QITs and five with a low QIT functionality were sampled (Table 1).

**Table 1:** Sample characteristics of health workers and health facilities, across high and low QIT functionality.

			QIT functionality		
	Sample characteristics		Low	High	All
**Health worker**	**Cadre**	Clinicians (N)	3	2	5
		Nurses (N)	4	6	10
		Medical attendant (N)	1		1
	**Median years in profession (N)**		16	10	10
	**Median years in current health facility (N)**		4,5	9	6
**Health facility**	**Type**	Hospital (N)		3	3
		Health centre (N)	3		3
		Dispensary (N)	5	5	10
	**Median number of health workers** (N)		4	3,5	3
	**Highest cadre in health facility is a Clinical Officer or above** (% of all sampled health facilities)		63	100	80

Respondents were contacted in advance, by telephone where possible, else by going to see them in the health facility. They were asked if they would be willing to participate and when a suitable time for the interview would be. At the time of the interview, informed written consent was then sought from all respondents. Interviews were held in a private area of the health facility. Interruptions to allow respondents attending to their patients were made and for this reason, some interviews took place over a longer period. The median effective interview time was 1 hour 12 minutes (range 1 hr 3 min to max 2 hrs 7 min). Interviews were co-conducted in Swahili by the first (UB) author together with a female Tanzanian social scientist (FH). UB is a Swedish female medical doctor with a master’s degree in Public Health who had two years’ work experience as a clinician and program manager in rural Tanzania and could easily establish rapport with the respondents. This study was the final study of her PhD. FH had experience from several qualitative studies in southern Tanzania. Both are fluent Swahili-speakers and minimal translation into English by FH to UB was done during the interviews. Neither of the interviewers was known to the respondents beforehand.

The interview guide (S3 File) was adapted after the first interviews as some questions did not yield sufficient response and to reflect new ideas emerging during the data collection. The very first interview was considered a pilot and not included in the analysis for this study. While the number of interviews was pre-determined, it was felt that saturation in the interview material was reached before the last few interviews were conducted as no or little new information emerged. No repeat interviews with respondents were done. The interview guide was divided into two parts, of which only the second part was analysed and reported on in this study. Results from the analysis of the first part have been reported on elsewhere [29] and are referred to in the discussion and interpretation of results.

Interviews were audio recorded and transcribed verbatim. Subsequent translation into English was conducted with careful attention to ensure the preservation of the original meaning. During analysis, any sections with unclear meaning were compared with the Swahili transcripts for clarification. Transcripts were not shared with respondents for validation.

### Data analysis

Data analysis was led by the first author (UB) with review by three of the co-authors (AP, AB, and CH) at different stages of the analysis. Qualitative content analysis was conducted by applying a theory-driven deductive approach [30] using the i-PARIHS framework as a lens. All transcripts were sorted into content areas correlating to the four i-PARIHS constructs [26]applied as themes for the analysis, then pasted into Microsoft Excel spreadsheets. Sorted paragraphs were further analysed deductively applying the characteristics of each i-PARIHS construct [28]as categories. The text was condensed into meaning units with codes applied which were grouped into sub-categories. Not all characteristics of each i-PARIHS construct were represented in the transcripts and the same sub-categories were sometimes reflected in more than one category resulting in some categories being merged. The emphasis during data analysis was on the themes of innovation and facilitation, and less on the recipients and context as these themes have been explored in detail in a previous paper [29].

## Results

Table 2 outlines an overview of the results along the main themes, corresponding to the four i-PARIHS constructs of innovation, recipients, context and facilitation. Detailed results for each theme are described below.

**Table 2:** Results.

Theme	Category	Sub category
***INNOVATION***	**Clarity**	Purpose to improve mother and child health
		Working on all improvement topics
		Can’t remember improvement topics
		PDSA-cycles to check problems and take action
		PDSA-cycles not easy to understand
		We record on run-charts every month
	**Degree of fit**	It is within our responsibilities
		EQUIP brought what we needed

	**Degree of novelty**	New strategies for old problems
		Mothers should stay longer
		Never used a run-chart before
	**Relative advantage**	Now we know our performance
		Now aware that we are able to help
		Work is easy when a mother is equipped
	**Observable results**	Mothers come
		Complications are decreasing
		Now we document
		
***RECIPIENTS***	**Motivation**	This job is my heart I wanted to help the community
	**Values and Beliefs**	We have to commit ourselves Community is not knowledgeable
	**Time, resources and support**	Working alone in a difficult condition We request but nothing is done
	**Collaboration and team work**	We cooperate Those who attend training give feedback
	**Power, authority and presence of boundaries**	I was posted For a nurse to prescribe is interfering Issues beyond our capacity I turn to wear the cap of a clinician
		
***FACILITATION***	**Participation and ownership**	We talk together
		They come to look at records
		They direct us
	**Integration and empowerment**	They are good in follow-up
		They remind you
		They gave us ways to fight the problems
		Good opportunity to learn from colleagues
		
***CONTEXT***	**Experience of innovation and change**	Projects often overlap Each project focuses on its activities Projects are helpful
	**Absorptive capacity**	Working alone There are many different projects

## 1. INNOVATION: Experience and understanding of the EQUIP intervention

### 1.1 Clarity

The clarity of the EQUIP intervention was reflected in sub-categories describing health workers’ perceptions of its purpose, their memory of improvement topics and understanding and use of the PDSA-cycles and run-charts (Table 2).

The purpose of EQUIP was perceived as improving maternal and child health by the majority of health workers although some described the purpose as providing training. Some health workers could not remember the improvement topics, whereas others were able to list them and described continuing to work on all of them during EQUIP.

There was a broad range of understanding and reported use of PDSA-cycles by health workers. Some health workers could describe the steps involved in a PDSA cycle and how to use it. A few described never having used a PDSA-cycle and some never having heard of it. Many health workers were unsure of its meaning and unable to explain the steps.

*I would say you plan something and do*, *and act and what is another; I forget one*. *S is for setting*, *you set*. *I have never used it*. (HW #15)

While a few health workers reported never having used a run-chart, the majority reported using them regularly, and the understanding was overall better than that of PDSA cycles. This understanding was reflected in the perceptions of their benefits and motivating effect described in the section on relative advantage (see below).

### 1.2 Degree of fit /with existing practice/

Health workers’ perceptions of the degree of fit between EQUIP and existing practice was reflected in two sub-categories: “it is within our responsibilities” and “EQUIP brought what we needed”.

The majority of health workers perceived that what EQUIP introduced was already within their responsibilities, indicating a high degree of fit with existing practice. Several health workers mentioned that, as opposed to programs dealing with one area of care, for example Prevention of Mother to Child Transmission (PMTCT), EQUIP dealt with all mothers regardless of HIV status. This more comprehensive approach was experienced as something positive.

*But for EQUIP all mothers*, *whether she is positive or negative, must be touched by EQUIP […] the same mother included in PMTCT, if you didn’t provide proper care she can die from other things apart from HIV and at the same time, the mother who is HIV negative, if you don’t provide proper care you may lose her due to other things and not HIV. Therefore other programs are for that, but …..I would say EQUIP is dealing with daily activities and touch every mother. (HW #16)*

Improvement topics were part of everyday practice, and health workers described having worked on these before. In some instances, the same problems as those identified during EQUIP had been the focus of previous improvement efforts; a prominent example of which was the high infection rates after Caesarean sections in the District hospital. The tools and support introduced by EQUIP were therefore welcome, also reflected in health workers’ appreciation of being reminded of skills learned during their pre-service training.

*We were using these postnatal strategies even in the past*, *but they were not like the way they are now*. *[…]*. *The significance and sensitization of postnatal care have increased*. (HW #3)

### 1.3 Degree of novelty

Although the perceived high degree of fit could be viewed as the opposite of novelty, health workers expressed that they had become aware of “new strategies for old problems”. One of these was the involvement of fathers during Antenatal care, to increase birth preparedness, which was expressed as positive by several health workers. The importance of mothers staying longer in the health facility after childbirth was also described as new knowledge, something that health workers were not aware of before.

The use of run-charts was a new tool for some health workers who described never having used it before EQUIP.

### 1.4 Relative advantage

Health workers’ experiences of EQUIP’s relative advantage compared to standard practice included knowing one’s performance, being able to provide better care to mothers who bring equipment and an increased awareness of being able to provide care despite challenges such as lack of drugs.

Knowing one’s performance through the use of run-charts was experienced as motivating by many health workers; they were perceived as help to evaluate one’s work, to know when to celebrate successes and when and where to put in more effort.

*You can assess yourself the way you have given services by looking at what you did the previous month and where you are now*. *I ask myself if I have been improving or not; have I delivered better services this month or not*? *What can I do so that I offer better services*? (HW#9)

Health workers also experienced the increased birth preparedness among mothers, perceived as an effect of EQUIP, to make their work easier and that it enabled them to provide better care.

*[…] when they come they have everything ready, therefore it becomes easy to offer service*. *(HW #3)*

### 1.5 Observable results

Health workers had noticed several changes following the implementation of EQUIP. The majority of these were changes in mothers’ behaviour, described in the sub-category “mothers come”. Health workers experienced that more mothers were coming to deliver in health facilities and that more fathers were accompanying their pregnant partners for ANC visits and at the time of delivery. These developments were viewed as positive, even though more mothers coming to deliver meant more work.

*Things are good [since starting to work with EQUIP]*, *we get many mothers for deliveries*, *we only refer a mother after failing completely […] I feel very happy*. (HW #15)

The increase observed in mothers’ birth preparedness was also part of this, as was the observation that mothers who had delivered at home would now come for follow up in the health facilities.

Complications in mothers and newborns were perceived to have decreased since the start of EQUIP. As mentioned previously, this was particularly emphasised in the hospital where a marked reduction of septicaemia in mothers following caesarean section was achieved.

Health workers also noted changes in their practice in that they had become better at documenting their work.

## 2. RECIPIENTS: Characteristics of health workers involved in quality improvement teams

### 2.1 Motivation, values and beliefs

When asked about the reasons behind their choice of profession, the majority of health workers expressed a strong internal motivation, illustrated in the sub-categories “this job is my heart” and “I wanted to help the community”. They described feeling encouraged when being thanked by pregnant women and mothers, and a desire to follow up those who had been referred. While a few health workers perceived that community beliefs and behaviours caused the main problems they were faced with; health workers overall expressed a belief of needing to commit to providing good services. This could include working overtime, something which was sometimes needed in order to complete additional reports for projects like EQUIP.

*Because I love my work*, *I provide care until I feel satisfied*. (HW #15)

### 2.2 Time, resources and support

Health workers described often having to “work alone in difficult conditions". Examples given included not having colleagues available to help during emergency situations, of having to perform many tasks by yourself and of not getting enough sleep due to constantly being on duty in the small health facilities. One health worker had been stationed alone in a dispensary for three consecutive years.

*In 2006 I was transferred again, to [name deleted] health centre […] I think I worked alone for three years. Then I was transferred to this place in 2009 and there was a medical attendant who was working here and she was about to retire. After her retirement, I worked alone for about a year […] probably she [current colleague] will also get transfer…I can see it, even now she is normally away for two months or one and a half month as she is involved in a distance learning programme; that it is the way it is*. *(HW #5)*

Health workers also described a sense of limited support from the district health officials, reflected in the category “we request but nothing is done”. This was not mentioned in relation to the EQUIP facilitation, but in general. Although they would convey their requests for assistance, they would not always receive this in a timely manner.

We do not understand what is being discussed there [at the district] because once we speak here that is it [nothing happens] (HW# 3)

### 2.3 Collaboration and team work

The category “we cooperate” reflects a sense of good cooperation among health workers and perceptions of helping each other when needed. Examples included direct patient care, where health workers would help each other to manage a difficult case such as PPH, distributing tasks when enough health workers were present, or sitting together to plan and make requests for drugs and equipment.

We cooperate, together we check what we don't have and we request together (HW#6)

The team work was also reflected in a few health workers expressing that colleagues who had attended training would report to the others about what they had learnt when they came back.

### 2.4 Power, authority and presence of boundaries

Health workers’ power and authority was also expressed as limited. “I was posted” was frequently described as the reason for working in the district or a particular health facility. Many issues, such as the frequent lack of drugs and equipment, were experienced as beyond health workers’ influence. Informal “task sharing” was illustrated by health workers perceptions of having to take on the role of higher cadre health workers.

*I'm a medical attendant [lower cadre health worker] but I have to do deliveries. I can’t tell a patient that the midwife is not around so it is impossible I must receive the patient*. *(HW #14)*

While this informal task sharing was necessary, lower cadre health workers described that their higher cadre colleagues sometimes perceived this as them interfering.

## 3. FACILITATION: Perceptions of EQUIP mentoring and coaching and learning sessions

### 3.1 Participation and ownership

Health workers’ sense of participation and ownership in implementing EQUIP was mixed, reflected in the sub-categories “we talk together”, “they come to look at our records” and “they direct us” (Table 2).

Some health workers experienced a high level of interaction and engagement with EQUIP, described as sitting and talking together with EQUIP mentors, of being asked questions and of being able to provide one’s views.

*We sit and discuss where we are*, *what to do*, *and way forward*. (HW#9)

A few health workers emphasised EQUIP mentors coming to look at the records in the health facility as a core activity, reflecting a more passive stance with a limited sense of ownership.

“They direct us” reflect a guiding role of the EQUIP mentoring and coaching which was mainly experienced as helpful. Health workers described being provided with solutions but felt that these were appropriate for their circumstances.

*[…] we were instructed by the people of EQUIP […] we didn’t have an idea on what we should do*, *but they are the ones who came to advise us […] we found out that the idea was suitable. (HW #8)*

### 3.2 Integration and empowerment

The extent to which health workers experienced the facilitation in EQUIP as integrated and empowering was reflected in four sub-categories; “they are good in follow-up”, “they remind you”, “they gave us ways to fight the problems” and “good opportunity to learn from colleagues”.

Many health workers articulated satisfaction, often unprompted, with the frequency and content of mentoring and coaching visits in individual health facilities. These were experienced to be integrated and health workers emphasised the iterative quality of the frequent follow-up and reminders.

*For example like yesterday we were there [at the learning session] and we were given other objectives*. *After two weeks they [EQUIP mentors] will come to visit and to see if we are implementing or not*, *if not why*? *And if we implement do we do that correctly*? *If there is any limitation*, *we are reminded how to do in order to succeed*. *They do not abandon us these people*. *(*HW #6)

While routine visits for supervision from the district to lower level health facilities would sometimes be experienced as negative, a positive change was felt following the implementation of EQUIP.

*Before*, *I was very scared when you heard about supervision [from the district]*, *you felt like running away because when they came here they complained […] but when they come [EQUIP mentors] […] the supervision perspective has changed […]*, *it’s very polite*. (HW#7)

Health workers expressed feeling empowered by the training provided by EQUIP; having increased their skills in problem-solving and enabling them to do things they couldn’t do previously.

*The thing which makes me really happy is the strategy that if something is missing in your facility–do not just sit*. *Just try to go to another facility*, *maybe you will find it*. (HW #17)

Learning sessions were mainly discussed by health workers when prompted. Experiences were overall positive with the opportunity to discuss and learn from others expressed as something good.

*Your challenge may differ from others or their challenges may not be yours*. *(#16)*

## 4. CONTEXT: Experience of innovation and change and absorptive capacity

### 4.1 Experience of innovation and change

The sub-category “projects often overlap” reflects the situation that health facilities in Tandahimba district were the recipients of several concurrent health programs run by research and non-governmental organisations; some of which like EQUIP focused on maternal and newborn care.

*I don’t know if there is a difference because, if you don’t know these organisations how they work, it is not easy to understand the difference*. *(HW #17)*

There could be as many as four external programs implemented alongside each other in some health facilities, experienced as a challenge in facilities with few, or sometimes only one, health worker.

Health workers were also not always clear about which programs did what and expressed the lack of integration between programs and their single-focus as something negative.

*The problem is that everyone [every program] is proud of they have […] but here we are not dealing with one thing*, *but many*. (HW #2)

At the same time, health workers experienced the presence of these various programs as positive and their activities as helpful. Specific examples included a positive feeling of being visited in one’s health facility, of receiving training and of being brought needed equipment.

*From my experience these projects are helpful (mmm). I think it is because that they come to visit time to time…I mean if it would be that you are just working without them to pass by, I think that would not be good (mmm) for sure projects are helpful*. *(HW#16)*

### 4.2 Absorptive capacity

Some health workers’ expressed that the presence of many different programs resulted in additional work load, especially in terms of documentation, as each project demands their own reporting.

*We normally fill the forms at the end of the month. We write so many reports and every project demand their report to be sent. […] It is a bit complicated in working performance, yes, it is a bit difficult*. *(HW#14)*

Health workers’ experiences of often working alone (described under the *Recipients* theme) and of being exposed to many different projects in the same health facilities can be interpreted as limiting the absorptive capacity, the degree to which health facilities have the capacity to incorporate new activities and implement projects.

## Discussion

In this qualitative process evaluation, we have explored the experiences and understanding among health workers of EQUIP, a district-wide collaborative QI intervention in rural Tanzanian health facilities. The results illustrate positive perceptions of several components such as a high degree of fit between the improvement topics in EQUIP and health workers’ priorities in every day practice and an appreciation of the intervention’s comprehensive, as opposed to a single-focus, approach. A clear motivating effect of using run-charts to monitor progress and an emphasis on the perceived importance of on-site mentoring and coaching visits to individual health facilities has also been found. Coupled with a high level of internal motivation to provide good care among health workers, in a context where many feel unsupported and lonely in their work as illustrated in a previous analysis of the same interviews [29], it is likely that these components have contributed particularly positively to the mechanisms of effect of the EQUIP intervention.

The identified lack of clarity around the use of PDSA-cycles has possibly led to this intervention component contributing less to mechanisms of effect in this setting. The context where health workers were exposed to overlapping projects implemented in the same health facilities may have a negative impact on the absorptive capacity to implement QI, reducing the EQUIP intervention’s potential effect.

The improvement topics which were selected during the EQUIP *prework* had purposefully been aligned to national guidelines and health workers’ routine work and responsibilities, something which was clearly noticed and appreciated. Similar findings were made in another study on training and health workers’ motivation in Vietnam [31]. The integrated approach of EQUIP through its focus on all mothers and newborns, was another expression of the high degree of fit, and health workers pointed out the tension of having to manage all patients and conditions while many programs would only focus on one or two.

While the reported use of run-charts varied, the expressions of their relative advantage and motivating effect were strong. The understanding of this QI tool was described as good, an important finding in a context characterised by many lower cadre health workers with limited pre-service training [32]. Run-charts as a standalone discrete intervention have been evaluated in high-income settings, also with positive effects on motivation, [33] and their motivating effect has also been reported by a QI initiative in a district hospital in Rwanda [34].

Contrary to run-charts, there was a wide range of understanding and reported use of PDSA-cycles, also found in a recent study investigating health worker perspectives of a QI intervention for institutional childbirth care in the same area of Tanzania [18]. In our study, health workers would only mention PDSA-cycles when prompted, suggesting that this intervention component was of subordinate importance to them. While EQUIP mentors consistently used PDSA-cycles, our findings suggest that the use of PDSA-cycles by QITs outside learning sessions or mentoring and coaching visits were limited. Conceptual understanding of the steps in the PDSA-cycle, especially by lower cadre health workers, may be challenging and one reason for its limited use [32]. However, a recent systematic review of the application of PDSA-cycles in high-income settings also found that application was inconsistent, negatively affecting this approach’s potential to improve quality of care [35]. An analogy can be made with the use of Partographs to monitor labour, where understanding and application by health workers is an ongoing challenge [36].

The positive experiences of the mentoring and coaching in EQUIP was an area of strong consensus among health workers, reflecting findings from previous studies where the level of senior support was identified as a critical determinant of health worker motivation and retention [37–39]. The supportive supervision typically carried out periodically in lower level facilities (health centres and dispensaries) in the Tanzanian and other Sub Saharan African health system has traits in common with the mentoring in coaching in EQUIP in that it provides *task-focused facilitation* [28, 40]. This kind of facilitation is characterised by a focus on certain goals/tasks, provides episodic contact and technical help and is usually of low intensity but with a high coverage [28]. There is however a wide range of roles that supportive supervision in the district is supposed to fulfil and, from a health worker perspective, it is often experienced as a control function rather than support [41, 42]. These findings were mirrored in a multi-country study where no correlation could be established between supportive supervision and quality of maternal care [43]. It is possible however that this finding was due to poor quality supervision or the way that this supervision was measured and defined. A recent study from two Tanzanian hospitals found that the quality of supportive supervision, together with other aspects of management, can have a significant impact on health worker motivation and performance [44]. The same was found in a Rwandan study on improving quality of Integrated Management of Childhood Illness [45], in a Nigerian study where significant improvements were achieved in service delivery for maternal, newborn and child care through clinical mentoring as a single component intervention [46], and in India where mentoring significantly improved the skills and knowledge of labour and delivery nurses [47]. Our results mirror these findings and emphasise the importance of good quality supervision, or rather mentorship, as an important mechanism of effect to improve the quality of maternal and newborn care in Tanzanian districts [48].

While the experience of learning sessions in EQUIP was positive overall, similarly to PDSA cycles, health workers only shared their experiences of them when prompted. Their perceptions suggest subordinate importance of these meetings compared to the mentoring and coaching visits to their facilities. This was also found in the study on health worker perspectives of QI in the same area of Tanzania mentioned above, and similarly from high-income settings [18, 49].

The context in which EQUIP was implemented had many challenges which have been reported on in detail elsewhere and which were likely a strong determinant of the limited measurable effect of the intervention [12]. What came out additionally in this study was the potential negative influence of other concurrent health programs implemented in the same district. Tandahimba had several partners operating health programs targeting mothers and newborns simultaneously. This is commonly found in Tanzania despite efforts put in place to coordinate programs and likely affects the health system’s absorptive capacity to incorporate change [18, 50, 51]. Health workers in our study felt ambivalent towards this presence of alternate agendas and approaches, which have also been found in other areas [52]. While the attention provided by external partners was experienced as motivating, health workers were not always able to distinguish between programs and sometimes felt overburdened by their demands. A recent review of the plethora of varying QI methodologies in the Tanzanian setting also pointed out the potentially harmful effects of this lack of coordination; including duplication, inefficiency and a feeling of confusion among front-line health workers [16].

## Methodological considerations

While time and funding for this study did not make it possible to conduct interviews in all 32 health facilities in Tandahimba district, our purposive sample aimed to reflect the range of functionality of QITs, the level of health facilities and cadres of health workers. As such, we believe that the results illustrate health workers’ experiences of the implementation of EQUIP in a comprehensive way.

While the interviewers were not involved in the implementation of EQUIP, health workers may still have been subjected to a social desirability bias, potentially skewing results to be more positive or limit results to areas that health workers felt comfortable to talk about. Our study could not distinguish facilitation from EQUIP mentors from that from district mentors, as frequently health facilities were visited by the two together. EQUIP mentors, as opposed to district mentors, had dedicated time for their task which could have enhanced the health workers’ positive experiences.

While the i-PARIHS framework was applied as a lens for analysis, it was not used to develop the interview guide and the results therefore did not reflect all characteristics of the i-PARIHS constructs. As in any deductive analysis, it is possible that some information which didn’t fit the predetermined categories were excluded. Although the qualitative analysis was led by the first author, three more authors were also contributing and we therefore believe the risk of omitting significant information to be small.

At the same time, we believe that the use of i-PARIHS strengthened our analysis in that it relates to theory of the key constructs known to affect implementation and also links this to the underlying theory of collaborative QI as outlined in our schematic logic model. This analytical approach fills an important gap in the improvement and implementation science literature [53, 54].

This process evaluation was purposefully conducted from the perspective of health workers and other perspectives, such as those of the EQUIP mentors or district managers, was left out. Another limitation was also that it only considered the first stage of mechanisms of effect, i.e. the interaction between health workers and the EQUIP project, and left out the interaction between health workers and the mothers or patients. It is possible that certain components of the EQUIP intervention impacted positively on the first level of mechanisms but not the second, which we were not able to assess. The evaluation was also based on health workers’ accounts with no inclusion of observations that could have deepened the understanding of how the intervention worked.

Lastly, no measure or examination of the sustainability of the collaborative QI approach as implemented by EQUIP was included in this process evaluation; limiting the potential understanding of integration and application in routine health services over time.

## Conclusions

Quality improvement components that are well understood and experienced as supportive by health workers in everyday practice include strong alignment of health topics with local priorities, use of run-charts to monitor progress and mentoring and coaching in individual health facilities. Emphasising these components may enhance mechanisms of effect and result in more significant change; something which could also guide harmonisation between various methodologies and increase country ownership of QI approaches. Additionally, the implementation of concurrent, overlapping, external health programs in lower level health facilities should be limited.

## Declarations

### Ethics approval and consent to participate

Ethical approval to conduct the study was obtained from Ifakara Health Institute Institutional Review Board (Ref: IHI/IRB/No: 30–2012) and from the National Institute for Medical Research (NIMR/HQ/R.8a/Vo.IX/1704), both in Tanzania. Permission to conduct the interviews in sampled facilities was also obtained from the District Medical Officer in Tandahimba district. Health workers whose main responsibility was to provide maternal and newborn care were approached and scheduled for an interview if they agreed to participate. Informed written consent was obtained from all respondents at this time. Respondents were carefully informed about their right to refuse participation but all chose to participate.

## Supporting information

S1 File Detailed description of the EQUIP intervention in health facilities. (PDF)

S2 File Functionality assessment of Quality Improvement Teams in EQUIP. (PDF)

S3 File Interview guide. (PDF)

## Abbreviations

EQUIP: Expanded Quality Management Using Information Power
HW: Health Worker
i-PARIHS: integrated Promoting Action on Research Implementation in Health services framework
PDSA: cycles–Plan Do Study Act cycles
QI: Quality Improvement
QIT: Quality Improvement Team
