# Supplementary material for: Health workers' experiences of collaborative quality improvement for maternal and newborn care in rural Tanzanian health facilities: A process evaluation using the integrated 'Promoting Action on Research Implementation in Health Services' framework
Source: PLoS One. 2018 Dec 19;13(12):e0209092. doi: 10.1371/journal.pone.0209092 (PMC6300247; doi:10.1371/journal.pone.0209092)
Supplement: S1 File — (PDF) [file pone.0209092.s001.pdf]

S1 File. Detailed description of the EQUIP intervention in health facilities.

EQUIP was an initiated by a consortium of researchers from five universities; hereafter referred to as the *EQUIP implementing team* [17]. This team, with involvement from the Tandahimba Council Health Management Team (CHMT) and with support from an external QI consultant from South Africa, carried out a substantial amount of *prework* to enable the effective implementation of EQUIP. One of the first steps was to create a *Quality improvement charter*; a document based on World Health Organisation (WHO) guidelines and national policy documents which outlined priority areas for improvement, referred to as *improvement topics* where previous research proposed low implementation levels. These included e.g. increasing Partograph use to monitor labour and increasing Active Management of the Third Stage of Labour (AMTSL) to prevent postpartum haemorrhage. For each improvement topic, a package of *change ideas*, i.e. strategies to achieve improvement, was also created. Creating the QI charter and the change packages corresponds to the elements of *topic selection* and *theory building* in collaborative QI methodology [21].

The *recipients* of the EQUIP intervention were health workers of different cadres (clinicians, nurses and auxiliaries) providing maternal and newborn care in Tandahimba district, organised into *Quality Improvement Teams* (QITs). These teams were formed at the start of EQUIP in each of the 32 public and faith-based health facilities (one district hospital, three health centres and 28 dispensaries) providing maternal and newborn care [17]. Having multiple sites, ideally between 20 and 40 is a core element of the collaborative QI methodology, with the aim to share experiences and learning within the collaborative [21].

The *innovation* introduced by EQUIP was the use of *Plan-Do-Study-Act (PDSA) cycles* and *run-charts* to improve quality of care. PDSA cycles were used to aid structured problem solving in relation to prioritised improvement topics and to select change ideas to overcome those problems, as outlined

by the Quality Improvement charter. Run-charts were used to test and evaluate change ideas through measuring and tracking indicators reflecting the improvement topic. These aspects are integral to the *model for improvement* which is at the heart of collaborative QI and guided by the three questions: (1) What are we trying to accomplish; (2) How will we know that a change is an improvement; (3) What change can we make that will result in improvement [21, 52].

Facilitation in EQUIP, the process through which implementation of the innovation was enabled, consisted of *Learning sessions* for a cluster of health facilities and *Mentoring and Coaching* visits to individual health facilities. Both activities were carried out by an *EQUIP mentor*, a member of the EQUIP implementing team, together with a *district mentor*, a member of the CHMT. These mentors were responsible for all 32 health facilities at any one point during the project. Occasional support was also provided by the external QI consultant to prepare learning sessions and during mentoring and coaching visits.

Learning sessions were held on average three to four monthly. During these sessions, QITs were taught on how to use PDSA-cycles and run-charts. Improvement topics were introduced where health workers were updated on relevant clinical guidelines and district level data collected by the EQUIP continuous surveys [53] and presented with the help of *report cards*. Essential prework went in to preparing these sessions to ensure a *focused* approach, another core element of collaborative QI methodology where key messages are distilled for recipients' learning in order to avoid overwhelming them with new knowledge. As implementation progressed, these sessions were the forum for reviewing progress across health facilities. Experiences were shared between QITs, of which change ideas had been tested since the last learning session; what had worked well and where there had been challenges. The hypothesis of sharing experiences is that it contributes to constructive peer pressure through which is created a *tension for change,* necessary to achieve improvement [21]. Learning sessions are also a vital element to achieve *spread,* the seventh element of collaborative QI methodology, which ensures dissemination of the new knowledge created.

This tension for change was also induced through the mentoring and coaching visits which were carried out on average once a month, during so called *action periods* in between learning sessions. During these visits, QITs were mentored to apply PDSA cycles and use run-charts to test change ideas targeting the various improvement topics. Mentors would review progress with individual QITs and discuss the particular issues pertaining to the provision of maternal and newborn care in their facility.

The hypothesised mechanisms of effect of EQUIP were that health workers' knowledge and skills would increase in relation to the improvement topics and in implementing quality improvement using PDSA-cycles and run-charts. The same was hypothesised for their motivation and capacity to overcome local obstacles to maternal and newborn care provision would increase. The collaborative QI elements of focus and tension for change central are to these mechanisms.
