# Supplementary material for: Health workers' experiences of collaborative quality improvement for maternal and newborn care in rural Tanzanian health facilities: A process evaluation using the integrated 'Promoting Action on Research Implementation in Health Services' framework
Source: PLoS One. 2018 Dec 19;13(12):e0209092. doi: 10.1371/journal.pone.0209092 (PMC6300247; doi:10.1371/journal.pone.0209092)
Supplement: S2 File — (PDF) [file pone.0209092.s002.pdf]

S2 File. Functionality assessment of Quality Improvement Teams in EQUIP

An assessment of each Quality Improvement Team's (QIT) functionality, i.e. how much they had understood, interacted with and applied the EQUIP intervention, was made. This assessment was conducted by the EQUIP Health Facility Mentor, Dr Petro Arafumin (also a co-author on this study), with additional input from the district mentors. This assessment was conducted as a "one-off" towards the end of the intervention period for all health facilities through scoring seven aspects of every QIT. While this scoring was subjective, it was structured and based on in-depth knowledge of the QITs. The seven aspects included whether the QIT (1) held monthly meetings (yes/no), (2) demonstrated knowledge of new topic (yes/no/some), (3) demonstrated knowledge of PDSA cycles (yes/no/some), (4) demonstrated knowledge of work plan for all change ideas (yes/no), (5) had change idea for most recent improvement topic (yes/no/some), (6) had documented their QI work for the past month (yes/no/some) and (7) plotted run-charts correctly and up-to-date (correctly plotted and up-to-date/ correctly plotted but not up-to-date/incorrectly plotted/not plotted). A QIT functionality score (0-13) was constructed using these seven aspects and enabled a crude grouping of health facilities into high- (≥ 10) and low- (≤ 9) functionality groups. The score was then used to inform the sampling of health facilities for interviews to reflect both high- and low functionality QITs.
