# Supplementary material for: Health workers' experiences of collaborative quality improvement for maternal and newborn care in rural Tanzanian health facilities: A process evaluation using the integrated 'Promoting Action on Research Implementation in Health Services' framework
Source: PLoS One. 2018 Dec 19;13(12):e0209092. doi: 10.1371/journal.pone.0209092 (PMC6300247; doi:10.1371/journal.pone.0209092)
Supplement: S3 File — (PDF) [file pone.0209092.s003.pdf]

S3 File. Interview guide in English and Swahili.

Part 1

1. Can you tell us a little bit about yourself; how you decided to become a health worker and how and when you came to work in this health facility?

2. To start with, could you tell us a little bit about your work load during the past week?

3. Could you describe what usually happens during a delivery in this health facility?

4. Could you tell us about a recent situation where you felt you were able to give a mother or newborn good care/where everything went well?

5. Could you tell us about a recent situation where you felt you were not able to give a mother or newborn all the services that you wanted to give them/a situation where you would have wanted to do more?

6. Thinking about these two examples; what do you feel are the main problems facing health workers in providing maternal and newborn care in Tandahimba district?

7. Could you tell us about any projects that have worked in your health facility to improve care?

8. In what way have you participated in EQUIP?

9. How would you explain the purpose of EQUIP to a new colleague?

10. Do you see any changes in your daily work since EQUIP started? If so, please explain/give examples.

Sehemu 1

1. Unaweza kutuambia kuhusu wewe mwenyewe; vipi uliamua kuwa mfanyakazi wa afya; jinsi gani na lini ulikuja kufanya kazi katika kituo hiki?

2. Kuanza, unaweza kutueleza kidogo kuhusu mzigo wa kazi yako katika wiki iliopita?

3. Unaweza kueleza kile kwa kawaida hutokea wakati wa kujifungua katika kituo hiki?

4. Unaweza kutueleza kuhusu hali ya hivi karibuni ambapo uliona ulikuwa na uwezo wa kutoa huduma nzuri kwa mama au mtoto mchanga (ambapo kila kitu kimeenda vizuri)?

5. Unaweza kutueleza kuhusu hali ya hivi karibuni ambapo uliona hukuwa na uwezo wa kutoa huduma zote ambazo ulitaka kumpatia mama au mtoto mchanga / au hali ya hivi ambapo ulitaka kutoa huduma zaidi?

6. Kufikiria juu ya mifano hii miwili; je, unaona nini ni matatizo makubwa yanayowakabili wafanyakazi wa afya wakati wa kutoa huduma ya uzazi na watoto wachanga katika Wilaya wa Tandahimba?

7. Je, unaweza kutueleza miradi yoyote ambayo inafanya kazi katika kituo chako cha afya ili kuboresha huduma?

8. Jinsi gani umeshiriki katika EQUIP?

9. Jinsi gani utaweza kuelezea madhumuni ya EQUIP kwa mshiriki mpya?

10. Je, unaona kuna mabadiliko yoyote katika kazi zako za kila siku tangu umeanza kufanya kazi na EQUIP? Kama hivyo, tafadhali tueleze/toa mifano.

Part 2

11. If not already covered: Can you describe the topics you have been working on in your health facility during EQUIP?
    i. Probe: List of topics from EQUIP
    - (Which topic(s) are you working on at the moment?)

12. We would now like to go through each topic you have been working on:

a) Describe the change ideas you used to improve quality of care

b) In your opinion, have the change ideas been effective?
- If yes, why? How did they lead to an improvement?
- Can you give an example of how they have changed your work in the health facility?
- If no, why not? Why did they not lead to an improvement?

c) How did you conclude if the change idea was successful or not?
- Can you show us any run-charts or PDSA cycles from your work with EQUIP and explain what they show? How would you use it?

d) Had you ever tried these change ideas before working with EQUIP?
- If yes: When /in what context? What were the results?
- If no: Had you tried anything else to solve the problem? Please explain. What prevented you from trying these change ideas before?

Questions to ask at the end if not brought up by the respondent in previous answers:

13. During your work with EQUIP, have you ever seen a report card (show sample)?
    a. Can you explain what it shows?
    b. Have you used it in your work and in what way?

14. Have you attended any learning sessions/meetings with health workers from other facilities while working with EQUIP? If so, what was your experience? What did you learn from the other health workers?

15. Have you cooperated with volunteers during your time in EQUIP? What was your experience? What did you discuss with them?

16. Did you cooperate with the district team during your work with EQUIP – in what way?

Sehemu 2

11. Je, unaweza kueleza mada ya huduma (topic) ambazo umekuwa ukizifanyia kazi katika kituo hiki kipindi cha EQUIP?

ii. dadisi: MBK; kujifungua kituoni; huduma baada ya kujifungua;

12. Sasa, tungependa kusikia mada moja baada ya nyingine:

e) Tueleze mikakati ya mabadiliko uliyotumia kuboresha huduma.

f) Kwa maoni yako, mikakati ya mabadiliko imefanikiwa?

- Kama ndiyo, kwa nini? Jinsi gani imesababisha uboreshaji?
- Unaweza kutoa mfano ni jinsi gani mikakati ya mabadiliko imebadilisha kazi zako katika kituo chako cha afya?
- Kama hapana, kwa nini?

g) Jinsi gani umehitimisha kuwa mikakati ya mabadiliko imefanikiwa au hapana?

- Unaweza kutuonyesha PDSA cycles au takwimu yoyote kutokana na kazi yako na EQUIP? Tueleze maana yake. Una itumiaje?

h) Umewahi kutumia mikakati hii kabla ya kufanya kazi na EQUIP?

- Kama ndiyo: Wakati gani?? Nini yalikuwa matokeo yake?
- Kama hapana: Je, umewahi kujaribu njia yoyote kwa ajili ya kutatua tatizo? Tafadhali tueleze. Kitu gani kimekuzuia kujaribu mikakati ya mabadiliko kabla?

<u>Maswali ya kuuliza mwishoni (kama mhojiwa hakueleza katika majibu ya mwanzoni)</u>

13. Katika kazi yako na EQUIP, umewahi kuona kadi ya ripoti (kama hii – mwoneyshe sample)

a. Unaweza kueleza inaonyesha nini?
b. Je, Uumekuwa ukitumia katika kazi yako? Kwa njia gani?

14. Je, umehudhuria learning session/mikutano na wafanyakazi wa afya kutoka vitua vya afya vingine katika mradi wa EQUIP? Kama ndiyo, nini ilikuwa uzoefu wako? Kitu gani umejifunza kutoka kwa wahudumu wenzako?

15. Je, unewahi kushirikiana na wahudumu wa afya wa kujitolea katika mradi wa EQUIP? Kama ndiyo, nini ulikuwa uzoefu wako? Mlijadili kitu gani?

16. Je, ulishirikiana na wafanya kazi wa afya kutoka wilayani wakati wa kufanya kazi na EQUIP? Kwa njia gani?
